# A 2.5D Self-Training Strategy for Carotid Artery Segmentation in T1-Weighted Brain Magnetic Resonance Images

**DOI:** 10.3390/jimaging10070161

**Published:** 2024-07-03

**Authors:** Adriel Silva de Araújo, Márcio Sarroglia Pinho, Ana Maria Marques da Silva, Luis Felipe Fiorentini, Jefferson Becker

**Affiliations:** 1 School of Technology, Pontifícia Universidade Católica do Rio Grande do Sul, Porto Alegre 90619-900, Brazil; pinho@pucrs.br; 2 Hospital das Clínicas, Faculdade de Medicina, Universidade de São Paulo, São Paulo 05403-010, Brazil; anammarques@usp.br; 3 Centro de Diagnóstico por Imagem, Santa Casa de Misericórdia de Porto Alegre, Porto Alegre 90020-090, Brazil; 4 Grupo Hospitalar Conceição, Porto Alegre 91350-200, Brazil; 5 Hospital São Lucas, Pontifícia Universidade Católica do Rio Grande do Sul, Porto Alegre 90610-000, Brazil; 6 Brain Institute, Pontifícia Universidade Católica do Rio Grande do Sul, Porto Alegre 90619-900, Brazil

**Keywords:** magnetic resonance imaging, deep learning, artificial intelligence, volume segmentation, weakly supervised self-training

## Abstract

Precise annotations for large medical image datasets can be time-consuming. Additionally, when dealing with volumetric regions of interest, it is typical to apply segmentation techniques on 2D slices, compromising important information for accurately segmenting 3D structures. This study presents a deep learning pipeline that simultaneously tackles both challenges. Firstly, to streamline the annotation process, we employ a semi-automatic segmentation approach using bounding boxes as masks, which is less time-consuming than pixel-level delineation. Subsequently, recursive self-training is utilized to enhance annotation quality. Finally, a 2.5D segmentation technique is adopted, wherein a slice of a volumetric image is segmented using a pseudo-RGB image. The pipeline was applied to segment the carotid artery tree in T1-weighted brain magnetic resonance images. Utilizing 42 volumetric non-contrast T1-weighted brain scans from four datasets, we delineated bounding boxes around the carotid arteries in the axial slices. Pseudo-RGB images were generated from these slices, and recursive segmentation was conducted using a Res-Unet-based neural network architecture. The model’s performance was tested on a separate dataset, with ground truth annotations provided by a radiologist. After recursive training, we achieved an Intersection over Union (IoU) score of (0.68 ± 0.08) on the unseen dataset, demonstrating commendable qualitative results.

## 1. Introduction

Medical image segmentation is a complex yet crucial process within the realm of image analysis. It serves as the foundation for extracting and isolating specific regions of interest. Segmentation is important for conducting detailed quantitative analyses and providing valuable insights into various medical conditions and anomalies. The emergence of deep learning has revolutionized medical image segmentation by automating and refining this intricate process. These techniques, especially convolutional neural networks (CNNs), have shown remarkable capabilities in segmenting medical images with high accuracy and efficiency. Automation saves time and introduces reproducibility to the image analysis pipeline. However, important challenges regarding deep learning segmentation of medical images need to be addressed, including dataset scarcity and difficulty in segmenting 3D structures.

Manual annotations are inherently time-consuming because they require detailed classification of numerous pixels within each image. In medical imaging, this is even more challenging since the segmentation needs to be validated by an experienced professional. It is not feasible to have a clinician spend time curating these masks in clinical settings. Unlike image classification tasks, where annotating each image with a single class label is relatively straightforward, segmentation tasks require meticulous labeling of pixels to accurately outline regions of interest. These factors result in a shortage of annotated datasets for segmentation, which are typically smaller than datasets used for classification tasks.

One way to address the challenges of manual annotation and limited datasets is by using weakly supervised self-training methods [1,2]. These methods use weak annotations, such as bounding boxes, to start the training process. In the context of medical image segmentation, weak annotations can be seen as providing initial guidance by outlining the region of interest within bounding boxes while also recognizing the presence of background pixels. By focusing on the semantic information conveyed by most pixels within the bounding boxes, weakly supervised segmentation techniques effectively guide the training to prioritize relevant features while minimizing the influence of noise or inaccuracies associated with background pixels. The iterative self-training process enables the network to refine its segmentation predictions progressively, gradually improving segmentation accuracy without requiring extensive manual labeling efforts. The adaptive nature of weakly supervised self-training allows the model to learn from its predictions and iteratively enhance segmentation performance.

When segmenting volumetric medical images, an important decision involves the processing of the input. One method is to divide the 3D volume into 2D slices and train 2D models for segmentation based on intra-slice information. Another approach is to use the entire 3D volume as input. While 2D models offer faster computation and higher inference speed, they overlook crucial information between adjacent slices, hindering improvements in segmentation accuracy. Additionally, 2D segmentation results can be affected by discontinuities in 3D space, leading to suboptimal segmentation outcomes.

However, 3D CNNs offer a way to understand volumetric spatial information, but they have limitations. Because of the increased dimensionality, 3D CNNs require more significant computational resources and may be more susceptible to overfitting, especially when dealing with limited datasets. Additionally, the slice information that could have been used as multiple instances for model training is now condensed into a single input, exacerbating the challenge of training with limited data.

To bridge the gap between 2D and 3D CNNs, 2.5D segmentation methods [3,4,5,6] can be utilized. This approach aims to efficiently segment volumetric medical images by creating new architectures or implementing strategies to integrate volumetric information into 2D models. One way this approach combines the advantages of 2D and 3D methodologies is by focusing on a specific slice of a volumetric image while incorporating information from neighboring slices to generate a pseudo-RGB representation. This pseudo-RGB image effectively preserves 3D spatial relationships, enhancing the model’s ability to segment complex 3D structures accurately. By adopting a 2.5D segmentation approach, the segmentation techniques can leverage the computational efficiency of 2D models while capturing crucial spatial contextual information from 3D models.

Increasing the size of datasets and developing effective strategies for segmenting 3D structures are important for addressing a specific issue: carotid artery segmentation in brain magnetic resonance (MR) images.

The carotid arteries are located on each side of the neck and ascend to supply the brain. In axial medical imaging slices, they appear as circular or oval structures positioned laterally to the cervical vertebrae and medially to the sternocleidomastoid muscles. In T1-weighted MR images, the carotid arteries are surrounded by muscles with moderate signal intensity and fat with high signal intensity. This contrast helps distinguish the arteries, which typically have a lower signal intensity than the surrounding fat’s high signal intensity. However, blood flow within the carotid arteries can have variable signal intensity depending on the flow dynamics and the presence of any contrast agent.

Computed tomography (CT) and ultrasound are commonly used for and are important for carotid artery studies. However, MR images provide superior soft tissue contrast, enabling detailed visualization of carotid artery walls and plaque composition. It also allows for three-dimensional (3D) imaging, offering comprehensive volumetric analysis and reducing the operator dependency commonly associated with ultrasound. MR imaging can simultaneously image adjacent brain structures, facilitating integrated neurovascular assessments crucial for understanding vascular health’s impact on brain function. Moreover, unlike CT, MR imaging does not involve ionizing radiation, making it a safer option for repeated imaging and use in vulnerable populations [7,8].

Carotid artery segmentation in brain MR images has several applications, particularly in molecular quantitative imaging. Accurate carotid segmentation allows for the extraction of image-derived input functions for analyzing the biokinetics of positron emission tomography (PET) radiotracers after aligning brain MR images with PET [9,10]. Additionally, the segmentation of MR images allows for quantitative volumetric analysis of the carotid arteries, enabling detailed assessments of vascular health and potential pathologies [11,12].

Segmenting carotid arteries in medical imaging is challenging due to several factors, including the small size of arteries, which can vary greatly between patients and under different conditions. It is difficult to create a single segmentation model that fits all cases. Carotid arteries are also located near other important anatomical structures in the head and neck, making it hard for segmentation algorithms to accurately differentiate them from neighboring tissue. Moreover, carotid arteries often have complex branching patterns and curves, making it challenging to track their path through multiple imaging slices and volumes. Therefore, algorithms need to be able to handle intricate and non-linear structures. Even small segmentation errors can have significant clinical implications, highlighting the necessity for highly precise and reliable segmentation methods. All these difficulties are exacerbated when the imaging technique is not optimized for vessel detection, as in non-contrast-enhanced MR images.

In this study, we developed a deep learning pipeline that utilizes a 2.5D approach combined with a self-training methodology for segmenting the carotid artery in brain T1-weighted MR images without contrast. The model achieved an Intersection over Union (IoU) score of (0.68 ± 0.08) on an unseen dataset, demonstrating commendable qualitative results. This approach augments the slices of the input instead of employing 2.5D techniques directly within model architectures [13,14]. We also address the challenge of carotid artery segmentation in brain MR images. Unlike conventional vessel analysis techniques that frequently utilize CT or ultrasound, our approach leverages the soft tissue contrast and three-dimensional imaging capabilities of MR, facilitating integrated neurovascular assessment and offering valuable insights into the interplay between vascular health and brain function.

## 2. Related Work

Methods utilizing convolutional neural networks (CNNs) have shown effectiveness in automated and semi-automated vessel segmentation in MR images [15,16,17,18,19].

Elsheikh et al. [15] explored the application of CNNs for the automated segmentation of the cerebral vasculature in non-contrast-enhanced black-blood MR imaging (BBMRI) scans. Utilizing a hierarchical, multi-scale 3D CNN model, the researchers achieved a promising Dice similarity coefficient (DSC) of 0.72 on their test dataset. The model employed nested image patches with a U-net-type architecture, allowing for effective segmentation across multiple scales. The study highlighted the advantages of BBMRI over traditional time-of-flight magnetic resonance angiography (TOF-MRA), including reduced flow-related artifacts and better stent-related signal preservation. However, they acknowledged the need for further optimization and expansion of the volume of interest to improve segmentation accuracy, particularly in complex intracranial pathologies.

Quon et al. [16] developed a deep learning model for real-time segmentation of intracranial vessels in pediatric patients using preoperative T2-weighted MR scans. A modified 2D U-net architecture achieved an overall DSC of 0.75. The model showed higher accuracy for patients with normal vascular anatomy (DSC 0.77) than those with lesions (DSC 0.71). The discrepancy was attributed to vascular deformations caused by tumors. Despite the impressive reduction in segmentation time (from hours to seconds), the small sample size and the model’s lower performance in patients with intracranial lesions were noted as significant limitations.

Shi et al. [17] developed an automated vessel wall segmentation method using a U-net-like fully convolutional network for quantifying MR vessel wall images in patients with intracranial atherosclerotic disease (ICAD). The method achieved DSC of 0.89 for the lumen and 0.77 for the vessel wall, showing strong agreement with manual segmentation. The study’s clinical application revealed significant differences in the normalized wall index (NWI) between symptomatic and asymptomatic patients, underscoring the clinical relevance of the segmentation method. While the results were promising, they emphasized the need for large-scale quantitative plaque analysis to promote the adoption of MR vessel wall imaging in ICAD management.

Samber et al. [18] investigated using CNNs for the automated segmentation of carotid arteries in MR imaging data. Using a dataset of 4422 axial T2-weighted MR images, they trained separate CNNs for segmenting the lumen and vessel wall, achieving DSCs of 0.96 and 0.87, respectively. The CNN-based segmentation showed excellent agreement with expert manual segmentations, evidenced by high Pearson correlation and intraclass correlation coefficients. Despite the need for human supervision to ensure consistency, the study showed the potential for integrating CNN algorithms into software platforms to streamline workflow and reduce the burden on radiologists.

Regarding weakly supervised segmentation and 2.5D approaches, Chen and Hong [19] introduced Scribble2D5, a novel approach that addresses the limitations of existing scribble-based methods by enhancing 3D anisotropic image segmentation. Unlike methods that suffer from poor boundary localization and are primarily designed for 2D segmentation, Scribble2D5 leverages volumetric data. It incorporated a label propagation module and a combination of static and active boundary predictions to improve boundary accuracy and shape regularization of the region of interest. Extensive experiments on public datasets for cardiac, tumor, and abdominal MR images demonstrate that Scribble2D5 significantly outperforms current state-of-the-art scribble-based methods, achieving performance comparable to fully-supervised approaches. However, this method was not tested for segmenting MR vascular imaging.

Overall, these studies highlight the potential of CNN-based approaches for vascular segmentation and the use of weakly supervised segmentation in MR images. Automatic segmentation techniques significantly reduce segmentation time, have high accuracy comparable to expert manual segmentations, and are applicable across various vascular conditions and imaging modalities. However, common limitations include the need for larger and more diverse datasets, the variability in performance across different patient subgroups, and the necessity for human supervision in some cases. Future research should address these limitations to enhance automated segmentation techniques’ robustness, generalizability, and clinical applicability in brain vascular MR imaging, especially for sequences that are not optimized for vessel detection.

## 3. Materials and Methods

### 3.1. Datasets

We utilized 42 brain T1-weighted MR volumetric scans sourced from four distinct datasets (10 scans from Zareda et al. [20], 10 scans from Van Schuerbeek, Baeken, and De Mey [21], 10 scans from Koenders et al. [22], and 12 scans from OASIS 3 [23]) to train our model. The first three datasets are defaced; only the last dataset did not go under defacing.

**Figure 1:**
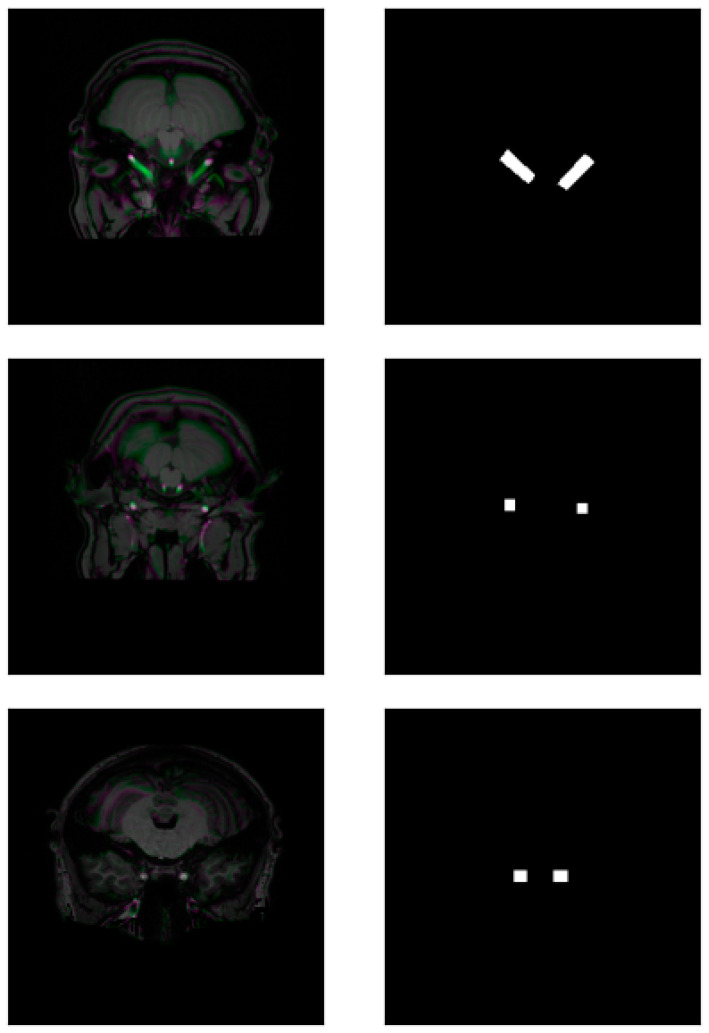

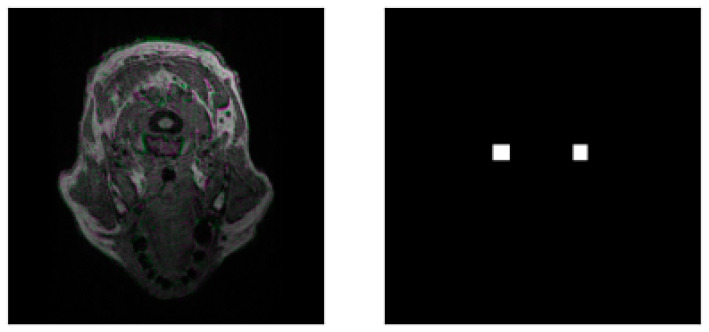
Pairs of 2.5D pseudo-RGB MR slices (**left**) and bounding boxes (**right**).

We meticulously delineated bounding boxes around the carotid arteries in each axial slice for each scan in our dataset. We performed an automatic 2.5D image processing for each slice, creating the pseudo-RGB images with the G channel being the target and the other channels being the neighboring surrounding slices (R is the slice below the target and B is the above one). At the end of the bounding box delineation process, we had 1869 pairs of slices and their corresponding masks. Visual representations of 2.5D pseudo-RGB MR slices (on the left) and their bounding boxes (on the right) can be seen in Figure 1.

We created a testing dataset to evaluate the model’s performance against a gold standard. The testing dataset was produced in a multiple sclerosis project. High-resolution structural brain T1-weighted MR images were acquired in a GE Healthcare Signa HDxT equipment of 3.0 T, using BRAVO^TM^ sequence, with a repetition time of 2400 ms, echo time of 16 ms, 220 mm field of view, with 1 mm isotropic voxels. MR images have an array of 240 × 240 × 196 pixels, with 16 bits per pixel.

**Figure 2:**
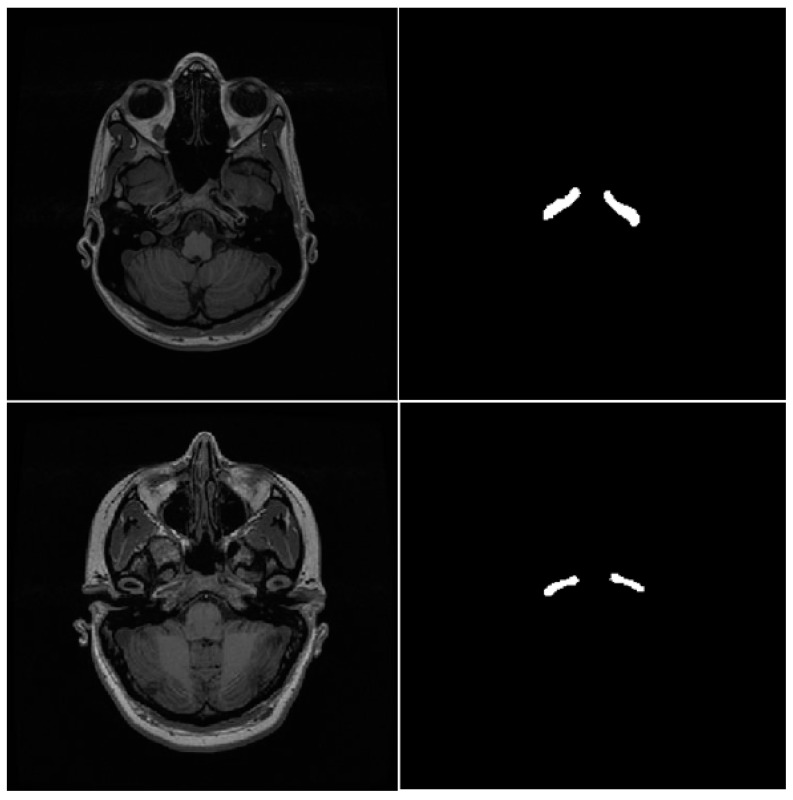
TR1-weighted MR slices and validated annotations of the carotid arteries.

MR scans corresponding to 35 individuals (age 30 ± 8 years) from the first visit were used to build the gold standard. The carotid arteries were visually identified and manually segmented by an experienced medical physicist. We constructed deformable two-dimensional polygons for all scans containing the left and right carotid slice per slice. An experienced radiologist validated each polygonal region, making corrections and modifications. After reviewing and correcting these polygons, we applied a binary transformation which converts the images into binary masks. We built pairs of images containing the original MR slice and its corresponding segmentation. This process allowed us to obtain 948 original pairs of MR slices and masks. Figure 2 shows examples of pairs of T1-weighted MR slices and corresponding carotid artery masks in the testing dataset.

### 3.2. Preprocessing and Data Augmentation

For the image preprocessing steps, we reduced the bit depth from 16 bits to 8 bits per pixel and normalized the pixel values by dividing each pixel by 255. Additionally, we ensured that the voxels in the 3D images were isometric (1 × 1 × 1 mm^3^). We hypothesize that not adjusting for bias inhomogeneities, spatial localization of brain structures, and parameters from the acquisition and reconstruction processes might aid in generalizing our models.

**Figure 3:**
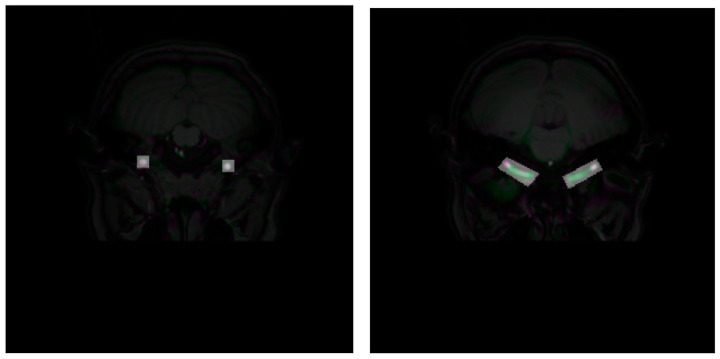
Overlap of the bounding boxes in different sections of the carotid arteries. The bounding boxes in the C2–C3 portions (**right**) have a larger area than those in the C1 portion (**left**).

The carotid arteries have a distinct shape distribution in the slices: they appear as smaller clusters in slices corresponding to the height where the vessels are classified as C1 or C4. In contrast, they appear as larger, cylindrical-like pixel clusters in slices corresponding to the height where the vessels are classified as C2 or C3 [24]. Figure 3 shows the overlay of bounding boxes to each type of carotid artery shape.

**Figure 4:**
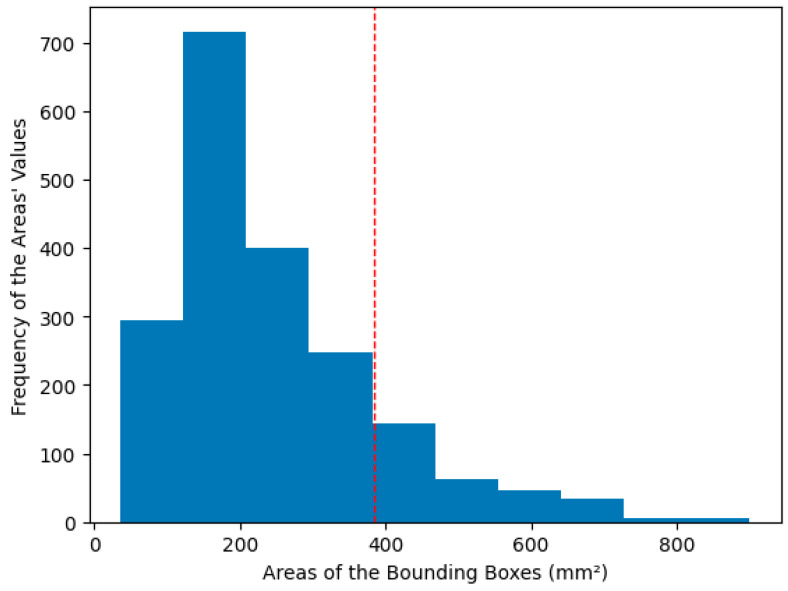
Histogram showing the frequency of bounding box areas in the training data. Lower areas are prevalent. The dotted line represents the mean + 1 standard deviation.

Generally, the bounding box areas covering the C2 and C3 regions of the arteries are usually larger, although this does not occur in most slices. Figure 4 shows the distribution of the areas of the bounding boxes in the carotid artery slices.

We were concerned that simply augmenting the data randomly would cause the models to focus only on the more common carotid shapes. To address this, we split our dataset into two parts. The first part consisted of images with masks with an area below the mean plus one standard deviation of the area (small area dataset). In contrast, the second part consisted of images with masks above this threshold (big area dataset).

We applied data augmentation to the large area dataset to increase its size by seven times and to the small area dataset to increase its size by 1.3 times. Overall, this augmentation doubled our entire dataset, and we applied this procedure throughout all the training rounds. We augmented the training dataset by applying random transformations to the images with a certain probability P. We used rotations (15° maximum, *p* = 0.9), horizontal and vertical inversions (*p* = 0.5), contrast modifications (*p* = 0.8), gamma (*p* = 0.5), blurring (kernel 3 × 3 and *p* = 0.05), Gaussian noise (*p* = 0.05), and shifts and zooms (*p* = 0.5). As we apply data augmentation techniques to the slices, we also apply the same modifications to the masks. The difference is that we use nearest-neighbor interpolation to preserve the masks’ binary values.

### 3.3. Model

The architecture we use is based on U-net [25]. The input consists of pseudo-RGB slices with 240 × 240 × 3 pixels. The process involves applying two padded 3 × 3 convolutional layers with a stride of 1, followed by a Parametric Rectified Linear Unit (PReLU) [26] and a 2 × 2 average pooling operation to downsample the data. Each layer has half the dimensions while doubling the number of feature channels. The final fifth level consists of two 3 × 3 convolutional layers, each with 1000 filters.

To restore the original image dimensions, we increase the resolution of the feature maps and combine the corresponding feature channels from each layer in the encoding phase of processing. This is followed by applying the PReLU function. The last layer involves a 1 × 1 convolutional operation that decodes the feature vector, generating a probability prediction for each pixel using a sigmoid activation function. We then apply a threshold to the probabilities at 0.5 for the final pixel classification.

**Figure 5:**
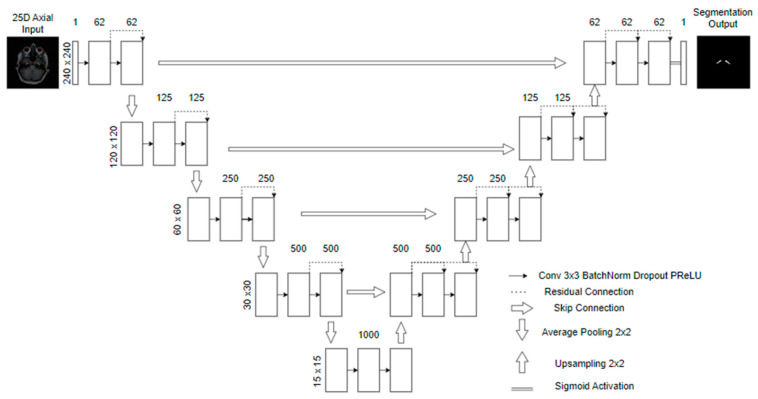
A variant of the U-net architecture with residual connections (Res-Unet).

We apply regularization to the model by incorporating batch normalization [27] and dropout [28] (*p* = 0.6) operations. We also include residual connections between the convolutions to retain features from previous layers by adding them to the newer features while creating new paths for gradient updates [29,30]. Figure 5 shows a scheme for the model.

We have implemented a learning rate schedule using an exponential decay function. This schedule gradually reduces the learning rate over time to aid the model in converging more effectively. We initiated the learning rate at 0.0001 and configured the decay steps to 253 with a decay rate 0.96.

We trained the models using the Adam [31] optimizer with a specified learning rate schedule. Since this is a segmentation problem, we required a loss function that could prioritize the foreground pixels. Therefore, we employed the Dice loss function [32,33].

### 3.4. Mask Update Scheme

We adopted the following four-step pipeline to update the masks from the training:

- We first train five models with the bounding boxes as the target for the semantic segmentation using 5-fold cross-validation (Round 0). We stratify the fold so that each fold has the same dataset separation. The model is trained for a maximum of 100 epochs. The training stops if the network does not improve the validation’s mean Intersection over Union (IoU) [34] in 10 epochs. We also only save the best weights in the validation.
- We used the trained models in an ensemble (average of the five cross-validation models’ predictions) to perform the segmentation in all the training dataset images, including those used to train them. After, we post-process the predictions using an erosion morphological operation, with a disk of radius one as the structuring element. This operation was performed only during the first four training rounds to eliminate a bit more of the false positives that naturally occur because of the initial bounding boxes.
- Using each post-processed mask, we calculate the IoU for the bounding boxes: if it is above 50%, we use the prediction of the post-processed mask as a new mask. If not, we return to the initial bounding box as a mask. We calculate the IoU for each carotid, separating the images into two parts, evaluating the image for each artery separately, and concatenating the results.
- Finally, we multiply the resulting mask by the bounding boxes, erasing pixels outside them.

This pipeline was repeated for seven rounds of training. Each time, we evaluated the segmentation results by comparing them to another dataset, this time with the radiologist’s gold standard.

## 4. Results

Table 1 displays the segmentation results compared to the gold standard for each training round.

**Figure 6:**
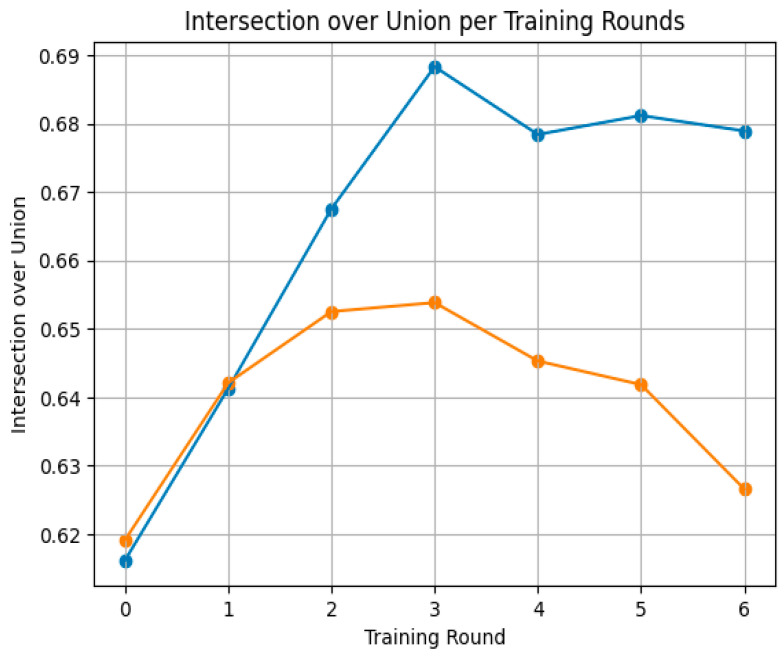
Comparison of the 2.5D approach (blue) with 2D slice segmentation (orange) using the same mask update technique.

The 2.5D approach increases the model’s performance, while maintaining it through the rounds of training, compared to using the same self-training strategy but with 2D slices, in which the performance worsened as the rounds progressed, as shown in Figure 6.

**Figure 7:**
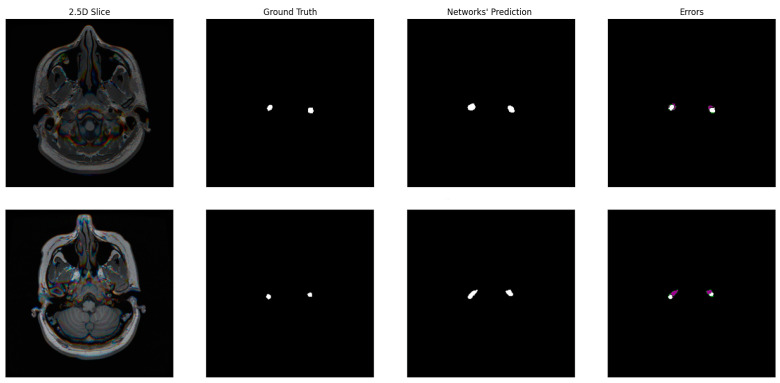

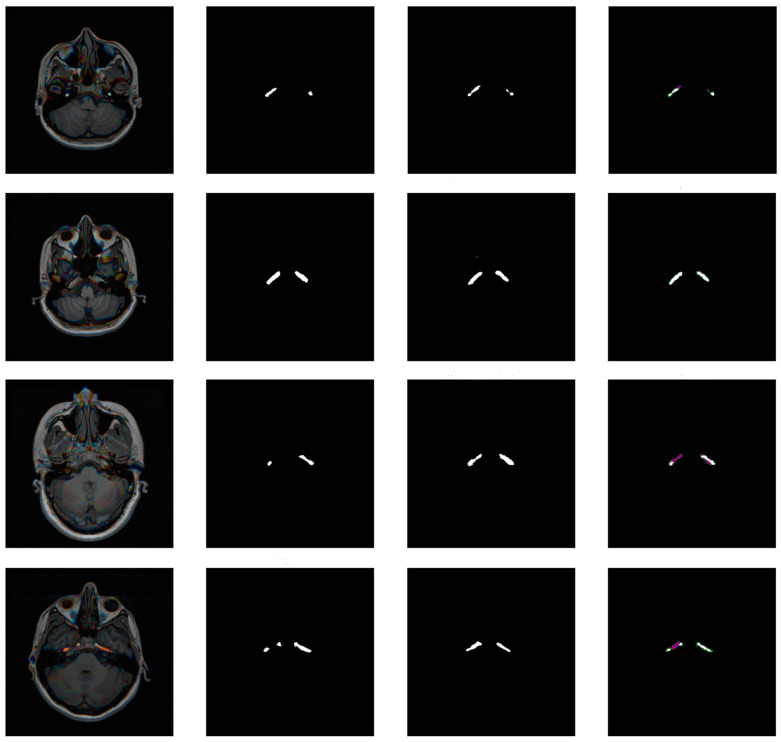
Collection of 2.5D pseudo-RGB slices and their predictions using the CNNs of the last round of training. The rightmost column shows the errors in the mask, purple representing false positive pixels and green being false negatives. True positives remain white.

The qualitative assessment of the generated masks revealed good segmentation results. Figure 7 shows examples of the segmentation results and errors using the model from the last round of training.

## 5. Discussion

Creating the bounding box dataset around the carotid artery region is straightforward in the slices of the volumetric T1-weighted brain MR images. This process is relatively simple compared to the pixel-level delineation of the arteries, which is significantly more time-consuming and requires the oversight of an experienced radiologist to validate the vessel’s segmentation. The bounding box approach allows for the faster creation of a larger dataset, which is beneficial for training more robust deep learning models.

In MR imaging, the dimensions of the carotid arteries, which typically range from 4–6 mm in diameter, are similar to the size of the 1 × 1 × 1 mm^3^ voxel dimension. This similarity poses a challenge when deciding whether to include the borderline pixels in the mask. With the weak annotations, we could recursively enable improvements in the mask with minimal manual intervention. This strategy reduced the dependency on labor-intensive manual annotations and improved segmentation performance systematically and progressively.

The 2.5D segmentation method enhances the results by incorporating information from the 3D structures of the arteries into a 2D technique. This combines the strengths of both approaches. Additionally, using adjacent slices as part of the input replicates how a human would analyze these images, considering the variation between slices to determine what is part of the vessel and what is not. This aspect is not equivalent to a purely 2D approach.

**Figure 8:**
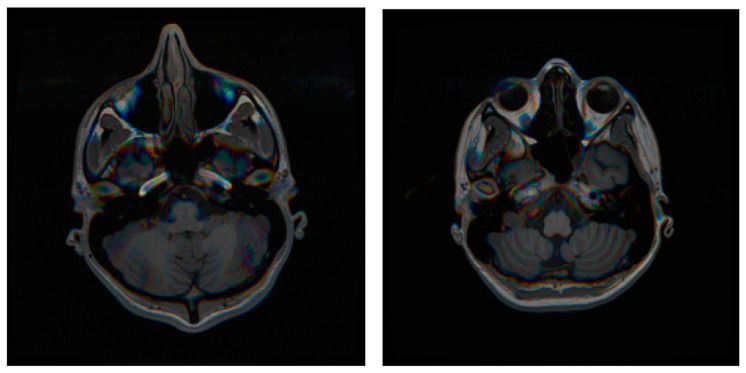
Differences in carotid arteries contrast in brain T1-weighted MR images in two subjects.

The similarity metrics (IoU and DSC) indicate that the model’s numerical performance was modest. This could be because brain T1-weighted MR acquisition protocols do not always provide good contrast for the carotid arteries. Images from the same dataset sometimes show different arterial contrasts, as illustrated in Figure 8, which explains the high standard deviation in IoU.

The challenge inherent in carotid segmentation was due to the small size of the carotid arteries relative to other brain regions. This size discrepancy rendered the segmentation task highly sensitive to errors, as even a few misclassified pixels could significantly impact the IoU and the DSC. When compared to some results related to the segmentation of brain arteries using similar data, our performance is lower (DSC = 0.72 [15], 0.75 [16], 0.89 [17], and 0.96 [18]). However, the comparison is not straightforward since these studies use vessel-specific sequences with contrast-enhanced sequences [15,16,17,18], whereas we utilized the standard brain T1-weighted MR sequences without contrast. Additionally, the study by Chen and Hong [19] used a 2.5D weakly supervised approach, but it was not employed for segmenting vascular MR images. Nevertheless, we can still make a high-level comparison of how our approach performs compared to similar studies.

Most of our predictions aligned with the ground truth, but the most common error was false positives. This is understandable, because we initially started with masks that naturally had false positives. One of the reasons we implemented erosion in the post-processing was to accelerate the removal of these false positive pixels surrounding the carotid arteries. These pixels were mainly located in the C2–C3 regions of the carotid arteries, which have larger areas and varying shapes, leading to significant changes from image to image. The carotid arteries regions have very few pixels for the foreground compared to the previous studies, which means that misclassifications of pixels make the relative error higher and the performance metrics lower. Nevertheless, the results show good qualitative agreement.

In our study, we used a variant of the Res-Unet model. This model provides opportunities for customization and improvement. In future research, integrating attention gates within the existing model architecture could enhance the model’s ability to focus on relevant features during segmentation [35,36]. The model architecture can also be replaced with more modern models, such as visual transformers or pre-trained architectures [37,38]. These alternatives offer advanced features and capabilities that could further optimize the segmentation process and yield even better results.

We can further improve our methodology by refining the mask update strategy. We can explore alternative methods to improve the initial segmentation guesses, using class activation maps as initial pseudo masks. Class activation maps use the model’s learned features to highlight regions of interest, potentially leading to more accurate and contextually relevant segmentation outcomes [39,40,41]. By incorporating these enhancements, we can refine our segmentation pipeline and achieve even higher accuracy and precision in carotid artery segmentation from brain MR images.

## 6. Conclusions

In conclusion, we developed a deep learning pipeline that addresses two critical challenges in medical image segmentation: the scarcity of annotated datasets and the loss of 3D information when using 2D slice-based approaches. By leveraging bounding boxes as initial masks and employing recursive self-training along with a 2.5D segmentation strategy, we enhanced the quality of carotid artery segmentation in brain T1-weighted MR images, achieving good performance on unseen data. Future studies could explore mask update schemes, experiment with various model architectures, and utilize larger image datasets to improve segmentation accuracy further.

## Figures and Tables

**Table 1:** Intersection over Union (IoU) and Dice similarity coefficient (DSC) of the ensemble prediction of 5 CNNs for each training round. Each round took around 2.5 h.

Round of Training	IoU	DSC
Round 0	0.616 ± 0.066	0.365 ± 0.169
Round 1	0.641 ± 0.073	0.426 ± 0.174
Round 2	0.668 ± 0.085	0.480 ± 0.197
Round 3	0.688 ± 0.085	0.526 ± 0.194
Round 4	0.678 ± 0.082	0.504 ± 0.198
Round 5	0.681 ± 0.080	0.512 ± 0.191
Round 6	0.679 ± 0.081	0.506 ± 0.193

IoU: Intersection over Union; DSC: Dice similarity coefficient.

## Data Availability

The data presented in this study are derived from both public domain resources and a private dataset. The images obtained from references [20,21,22] are all available on openneuro.org, while images from [23] are available at sites.wustl.edu/oasisbrains/home/oasis-3/. The annotated dataset used in this study is owned by the Brain Institute of Rio Grande do Sul and is only available for internal academic use. As such, it cannot be shared publicly due to institutional restrictions.
